# Relationships between tissue properties and operational parameters of a dental handpiece during simulated cavity preparation

**DOI:** 10.1177/1758736013483747

**Published:** 2013-03-25

**Authors:** Hongyan Sun, Andrew Lau, Young C Heo, Lianshan Lin, Ralph DeLong, Alex Fok

**Affiliations:** 1 The State Key Laboratory of Mechanical Transmission, Chongqing University, Chongqing, China; 2 Minnesota Dental Research Center for Biomaterials and Biomechanics, School of Dentistry, University of Minnesota, Minneapolis, MN, USA

**Keywords:** Dental handpiece, cavity preparation, feed force, rotational frequency, correlation coefficient

## Abstract

A preliminary study was conducted on the development of an intelligent dental handpiece with functionality to detect subtle changes in mechanical properties of tooth tissue during milling. Such equipment would be able to adopt changes in cutting parameters and make real-time measurements to avoid tooth tissue damage caused by overexertion and overextension of the cutting tool. A modified dental handpiece, instrumented with strain gauges, microphone, displacement sensor, and air pressure sensor, was mounted to a linear movement table and used to mill three to four cavities in >50 bovine teeth. Extracted sound frequency and density were analyzed along with force, air pressure, and displacement for correlations and trends. Experimental results showed a high correlation (coefficient close to 0.7) between the feed force, the rotational frequency, and the averaged gray scale. These results could form the basis of a feedback control system to improve the safety of dental cutting procedures. This article is written in memory of Dr Hongyan Sun, who passed away in 2011 at a young age of 37.

## Introduction

Drilling and milling with dental handpieces are common procedures in restorative dentistry. Handpieces are used in the removal of carious (dental decay) tissues, cavity preparation for the placement of restorative materials, tooth reduction for the construction of prostheses, *removal of old restorations*, cavity preparation in *jawbones* for the placement of dental implants, and root canal preparation in endodontic treatment. The dental handpiece is thus the most fundamental instrument used by the dentists. However, it is also the instrument most likely to cause tissue damage. The most common type of dental handpiece is an air turbine rotating at very high speeds, which range from 160,000 to 800,000 r/min. It is usually used “blind,” with no feedback, other than what the dentist can feel through the grip. Breaching into the pulpal space or sinus may occur due to overextension of the cutting tool, leading to permanent damage to tissues. Therefore, from a patient’s perspective, the development of an “intelligent” dental handpiece, which can identify the subtle changes in the cutting conditions and stop the cutting process automatically before overextension of the handpiece can occur, would be advantageous. In order to achieve this aim, it is essential to first study the operational parameters such as the cutting force and rotational frequency of the handpiece and then to analyze the relationships between the tissue properties. In practice, the air pressure supplied, the rotational frequency of the handpiece, and the cutting force applied are all highly variable and operator sensitive. Therefore, as a preliminary investigation, the dental milling procedure was studied using a laboratory setup whereby the feed rate could be controlled and where some of the operational parameters could be studied systematically.

Previous studies have provided many methods for monitoring the operational parameters in vitro.^1,2^ The measure of these parameters through laboratory-based investigations have been used primarily for comparative testing and handpiece selection,^3^ evaluation of their effect and that of the bur type on tooth damage,^4^ fault diagnosis of equipment,^5^ and the improvement of drilling performance through the design of suitable drill bit materials,^6^ and so on. However, there have been few studies considering the relationships between the operational parameters of a dental handpiece and properties of the tissues being cut. Such studies can be found in other research areas. For example, in their study of bone, Ong and Bouazza-Marouf^7,8^ find that the bone drilling forces have the potential to provide useful information about the strength of bone. The effects of system compliance and inherent drilling force fluctuation on the profiles of drilling force are explored, as well as the force variations in the drilling force between successive samples and the drill bit’s rotational frequency. It is shown that these effects have significant influences on the bone drilling force profiles and thus on the detection of drill bit breakthrough. In a series of studies, Brett and colleagues^9–11^ introduced an autonomous surgical robot for ear surgery. The robotic system monitors the force and torque transients at the drilling tool point and interprets these in real time. The relationship between the transients can be used to distinguish between the different states and phenomena, such as patient or tool movement, the approach to tissue boundaries, changes in tissue hardness and stiffness, and drill breakthrough. Using part of this information, it is possible to predict the critical breakthrough event before it occurs and automatically control the drill penetration with minimum protrusion.

## Materials and methods

### Sample preparation

**Figure 1.**
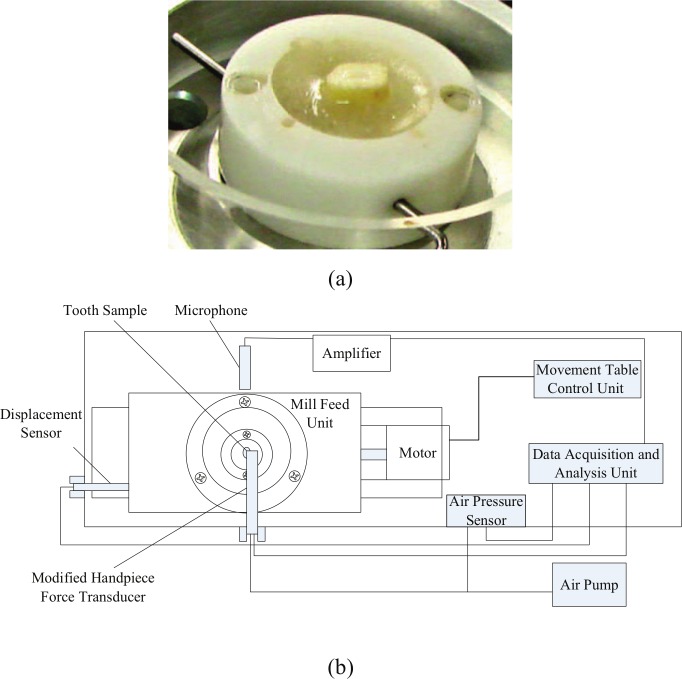
Apparatus setup: (a) tooth sample mounted in a Teflon ring using orthodontic resin and (b) system diagram of the experimental setup.

More than 50 bovine teeth were used in this experiment. Using an orthodontic resin, each tooth was vertically mounted in the middle of a Teflon ring, as shown in Figure 1(a). More than 5 mm of the coronal portion was placed above the resin surface. The tooth was then flattened to remove the top layer of enamel. All samples were stored in deionized water for 1 week prior to testing.

### Experimental setup

**Figure 2.**
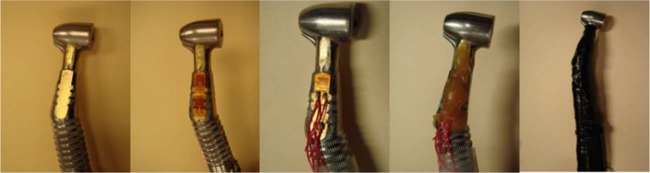
Procedure of modifying the handpiece into a force transducer.

As shown in Figure 1(b), the experiment was conducted on a linear movement table. A high-speed air-turbine dental handpiece (Midwest Quiet Air; Midwest Dental Supply, Des Plaines, IL, USA) was modified to become a force transducer whereby the feed force could be measured using full-bridge strain gauge circuits (Figure 2) during cutting of the tooth. Using a clamp, the handpiece was fixed to the bench on which the movement table was placed (see Figure 1(b)). A six-bladed, flat-end, straight fissure, tungsten carbide bur with a 1 mm head size and a 19 mm grip (US#57 FG Fissure Carb; Brasseler USA, Savannah, GA, USA) was used with the handpiece to cut the tooth samples. The emitted cutting sound was collected by a microphone (ECM8000; Behringer, Zhongshan, Guangdong, China) placed close to the handpiece. The microphone was connected to an amplifier (Tube MP, Rochester, NY, USA) with a Universal Serial Bus (USB) connection. A displacement sensor (LBB-375-PA-100; Schaevitz, Hampton, VA, USA) was used to measure the movement of the table, and a piezoelectric air pressure sensor DP-102A-N-P; Panasonic, Kadoma, Osaka, Japan) was used to measure the air pressure applied. Two computers were used in the experiment: one for controlling the speed of the movement table and the other for collecting the force, sound, displacement, and air pressure for subsequent data analysis.

### Experimental procedure

The experimental procedure was described as follows.

- The Teflon ring with the tooth sample was secured onto the movement table.
- The position and angle of the modified handpiece were adjusted so that the bur lay perpendicular to the linear movement table and positioned touching the tooth’s side surface.
- The displacement sensor was zeroed, and the data collection software was activated to begin recording the sound, feed force, displacement, and air pressure.
- The air and cooling water supplies to the handpiece were turned on, and the control program for the movement table was activated to drive the tooth sample toward the bur at a rate of 0.03 mm/s.
- During cutting, the air pressure was maintained manually at an approximate value of 29 lbf/in^2^.
- Three to four slots, measuring less than 1 mm deep, were milled over the top surface of the flattened tooth sample.
- Finally, the sample was placed in a micro-computed tomography (CT) machine (Metris XT H 225) to measure the gray scale values within each milled slot.

### Extraction of rotational frequency

Sound pressure was collected at a sampling frequency of 22,050 Hz. The sampling frequency was chosen to be greater than twice the maximum rotational frequency (7000 Hz), in accordance with signal processing theory for the avoidance of aliasing. The fast Fourier transform was performed for every 16,384 sound samples to extract the frequency spectrum. The main frequency within 1000–8000 Hz in the frequency spectrum was taken as the rotational frequency of the handpiece.

### Determining the gray scale values within a slot

**Figure 3.**
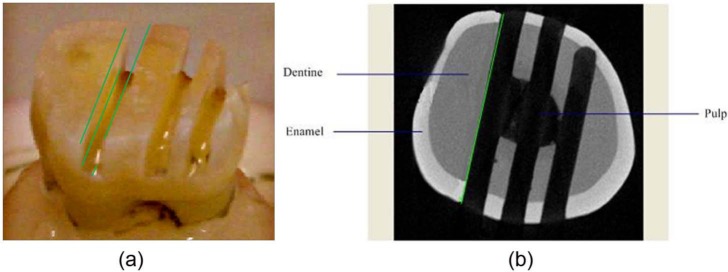
Images of the tooth with three slots: (a) parallel slots made on the flattened tooth sample and (b) micro-CT image of the horizontal cross-section. micro-CT: micro-computed tomography.

A tooth sample with three parallel slots made on its surface is shown in Figure 3(a). After micro-CT scanning (Figure 3(b)), the gray scale values along the length of each slot were determined. Each slot had four edges (shown as green lines in Figure 3(a)). By averaging the corresponding gray scale values of the four edges, an average gray scale value with respect to position was found.

## Results

Over the course of the study, over 150 slots were milled in more than 50 bovine teeth. All the millings showed similar results. For illustrative purposes, the results of one, randomly selected, slot will be shown. The relationships between some of the operational parameters and the tissue gray scale are shown in the following sections.

### Relationship between the feed force and rotational frequency

**Figure 4:**
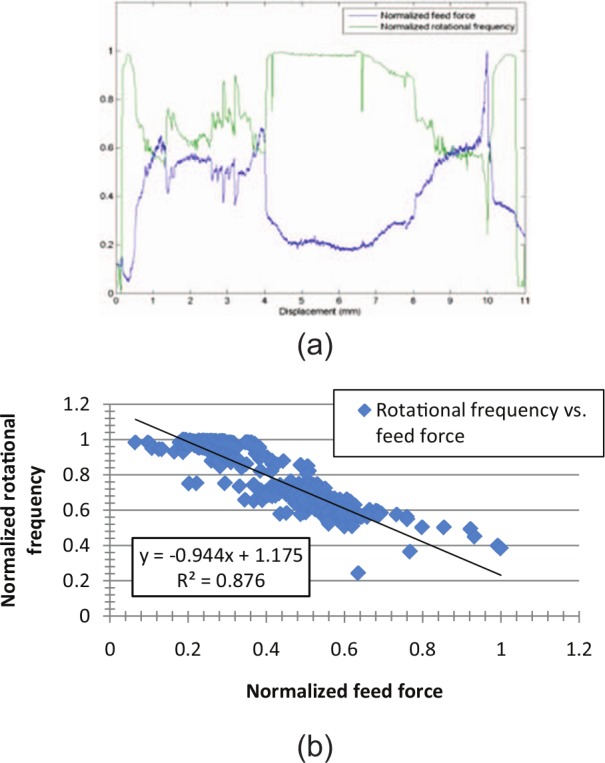
(a) Feed force and rotational frequency versus bur displacement and (b) feed force versus rotational frequency.

Figure 4(a) shows how the measured feed force and rotational frequency change as the bur milled out a slot in a tooth. The data have been normalized using the respective maximum values in the data sets. The profiles of the two parameters almost formed mirror images of each other: the higher the feed force, the lower the rotational frequency and vice versa. Higher feed force values (and lower rotational frequencies) were found at the beginning and end of the cutting, where there were also larger fluctuations in the profiles. Between 4 and 7 mm of displacement in Figure 4(a), the profiles were relatively smooth and steady. Figure 4(b) shows a clear linear relationship between the feed force and the rotational frequency. The correlation coefficient was close to 0.9 for all the slots milled in the experiment.

### Relationship between the feed force and the tissue gray scale

**Figure 5.**
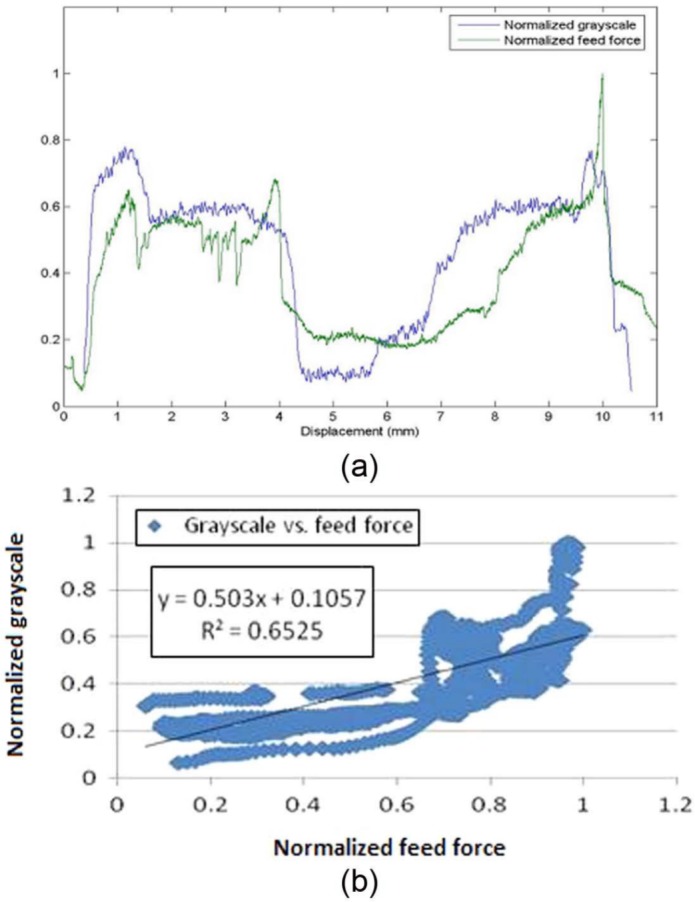
(a) Feed force and tissue gray scale versus bur displacement and (b) feed force versus average gray scale.

Figure 5(a) shows the normalized averaged tissue gray scale against the bur position. The normalized feed force is also plotted for comparison. In general, the gray scale of a material in a CT scan increases with its density. The different regions within the tooth can be identified from their gray scale values, that is, enamel (about 1 mm thick) on the outside and dentin on the inside. The middle region (4–7 mm), with the lowest gray scale value, was the region of the dentin just above the pulp where density was lowest. It can be seen that the feed force follows closely with the gray scale value of the tooth tissue. Thus, the feed force was highest when the bur was milling through the hardest enamel, and it was lowest when the bur was traversing the softest region above the pulp. The large fluctuations in the feed force occurred mainly in the denser dentin regions. There was a steady increase in the feed force in the beginning as the bur made its way into the tooth. This was not reflected in the gray scale profile, which showed a rather constant value for the enamel region. The fluctuations in the gray scale values were also smaller. Figure 5(b) shows the direct relationship between the feed force and the gray scale value of the tooth tissue. The coefficient of determination was close to or above 0.7 for all the slots milled in the experiment.

### Relationship between the rotational frequency and the tissue gray scale

**Figure 6.**
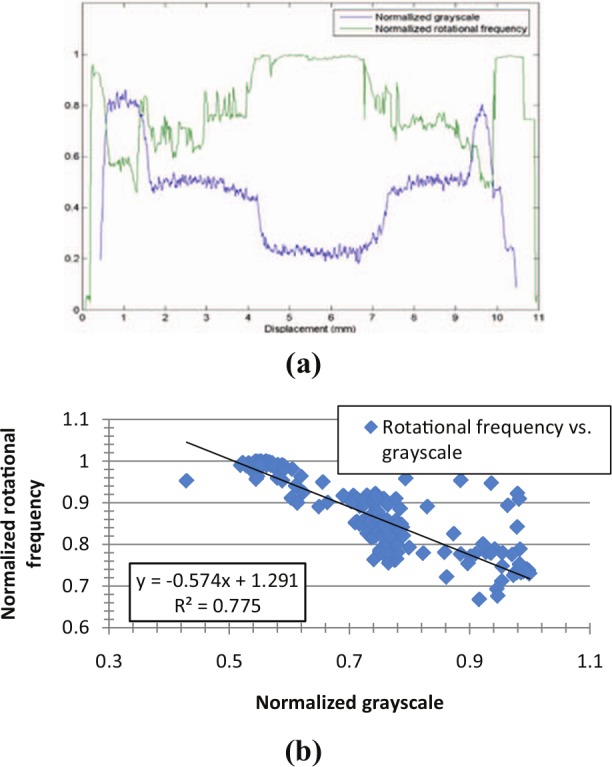
(a) Rotational frequency and averaged gray scale versus bur displacement and (b) averaged gray scale versus rotational frequency.

Figure 6(a) compares the changes in the rotational frequency of the handpiece with those of the tissue gray scale. Just as it did with the feed force profile, the rotational frequency formed near-mirror images with the gray scale or density: the higher the gray scale, the lower the frequency and vice versa. However, the rotational frequency shows much larger fluctuations in its profile. Despite this, the rotational frequency seems to match the tissue gray scale better than the feed force.

Figure 6(b) shows directly the relationship between the rotational frequency of the handpiece and the gray scale value of the tooth tissue. A coefficient of determination above 0.7 was found for these two parameters.

## Discussion

In this study that simulated the tooth reduction process via milling, some of the dental handpiece operational parameters were evaluated and their changes with the tooth anatomy examined. The monitoring of the operational parameters during the simulation indicated that changes in parameters such as rotational frequency and feed force could be used to identify changes in tooth anatomy.

The gray scale values obtained from a micro-CT scan reflect the ability of materials to absorb X-rays. It is therefore possible to use the gray scale values to distinguish between different tooth tissues. For materials with the same chemical compositions, their gray scale values will depend mostly on their densities. The denser a material is, the higher the gray scale value and vice versa. This can be seen in the results shown in Figures 3(b), 5(a) and 6(a). Both enamel and dentin are made of hydroxyapatite, but the former is denser than the latter. The plots of gray scale values versus position show three distinct regions with reducing gray scale values, that is, enamel, dentin, and pulpal space. The three values further indicate that each region is rather homogenous in density and does not fluctuate much other than in the intermediary junctions.

The feed force can be used to detect changes in the mechanical properties of the tooth tissues during milling of the tooth. It is expected that a softer, more compliant material would yield a lower feed force, while a harder, more brittle material would result in a larger feed force. The handpiece’s force response in Figures 4(a) and 5(a) supports this prediction. The force data showed a clear change in the feed force as the bur neared the expected dentino-enamel junction and the softer dentin above the pulp, with the harder enamel requiring a larger feed force than the softer dentin. The close relationship between the feed force and the gray scale values of the tooth tissues indicates the possibility of detecting material changes in real time during tissue removal through the dental handpiece. This makes the development of an intelligent dental handpiece with feedback control a promising prospect.

The third parameter explored in this study was rotational frequency. Rotational frequency of dental handpieces has been measured using methods such as mechanical tachometers,^12,13^ stroboscopes,^14–16^ optical counting devices,^17–20^ magnetic systems,^21–24^ and sound frequency detectors.^25^ Of these methods, sound frequency has been one of the easiest techniques to implement. Ball and Davidson^25^ have used both a close-proximity variable inductance vibration pick-up system and a separate microphone to measure air-turbine speeds during clinical use. Additional research by Morrant^26^ has found that the predominant sound frequency is equivalent to the rotational speed. Using sound frequency as an indicator of rotational speed, this study has measured the handpiece’s bur speed. The dental handpiece operates at a very high narrow frequency band which background noise cannot reach under normal conditions. Band filters can be used to remove background noises and harmonics. More than 150 cavities were milled, yielding results similar to Figures 4(a) and 6(a). The clear mirror-like responses between feed force and rotational frequency, Figure 4(a), suggest that evaluation of one parameter may serve as an indicator of the other.

It is recognized that this study used a rather idealized simulation to represent the actual clinical situation. For example, the bur was clamped and remained perpendicular to the occlusal surface during the movement. A constant and very slow displacement rate (0.03 mm/s) was also used. And only the cutting of healthy dental tissues was considered. In reality, the bur is not always perpendicular to the tooth surface. The rate of displacement is also faster and more varied. Furthermore, the bur is used to remove restorative materials, for example, in replacing old restorations as well as tooth tissues.

Tooth structures are very complicated, with tissue properties being highly variable with anatomical position.^26–29^ The large fluctuations in the feed force/rotational frequency produced when hard and brittle tissues were being cut made it difficult to detect, for example, the transition of the bur from enamel to dentin. It is conceivable that the magnitude of the parameters and their fluctuations could also vary with the cutting speed and air pressure, thus confounding the problem. In addition, the bur has a finite diameter of about 1 mm, which is comparable to the enamel thickness. This will also smear the transition, as it will take a finite distance for the bur to completely move from one region into the other. The gradual increase in the feed force as the bur first milled into surface enamel was probably the result of this gradual increase in the contact area between the tool and tissue. Furthermore, decayed dentin may be mistaken as the pulp because of its softness.

Another factor that may give rise to complications has to do with the fact that each handpiece will have its own compliance and cutting characteristics dependent on its internal mechanics. This would require a new calibration for each device. While the overall trends and responses between operational parameters should be similar, clinical conditions will further complicate the design of a control system for the device. The exact response of the handpiece will probably depend on the firmness of the dentist’s grip, the angle of cutting, the speed of motion, and the applied air pressure, which is often intermittent.

It seems hard to only use force and rotational frequency to identify real-time cutting tissues. It may therefore be necessary to combine multiple parameters and to perform more detailed signal processing in order to better identify the transitions from one region to the other. It may also be necessary to augment the mechanical data with positional data, supplied in real time by sensors attached to the handpiece and the patient, to help determine, for example, the position of the bur relative to the pulp, whereby early warnings to possible breaching can be provided.

This study recognizes the clinical constraints of the design of a high-feedback device. The first step has been to identify possible trends between the operational parameters. Additional research, such as looking beyond the main sound frequency and into transients and global positioning may provide important information. Despite this study’s limitations, there was a very distinct change in the feed force/rotational frequency profiles, which also became much smoother, when the softer dentin close to the pulp was being milled. This strongly suggests that an instrumented handpiece may be able to detect the approaching pulpal space before breaching occurs. Furthermore, this tool may serve as a means to safely and more precisely remove restorative materials without destroying healthy tooth tissues in the process of replacing old restorations.

## Conclusion

In this preliminary investigation, conclusions can be made that the feed force and rotational frequency of a dental handpiece operated with a constant air pressure and milling rate are directly correlated. Furthermore, the two parameters have a close relationship with the mechanical properties of the tissues being removed. It thus appears possible to detect changes in tissue properties during cavity preparation through the changes in these operational parameters.

## References

[1] Poole RL Lea SC Dyson JE Vibration characteristics of dental high-speed turbines and speed-increasing handpieces. J Dent 2008; 36: 488–493. 18468763 10.1016/j.jdent.2008.03.006

[2] Jantunen E A summary of methods applied to tool condition monitoring in drilling. Int J Mach Tool Manufact 2002; 42: 997–1010.

[3] Dyson JE Darvell BW Torque, power and efficiency characterization of dental air turbine handpieces. J Dent 1999; 27: 573–586. 10528975 10.1016/s0300-5712(99)00038-x

[4] Watson TF Flanagan D Stone DG High and low torque handpieces: cutting dynamics, enamel cracking and tooth temperature. Br Dent J 2000; 188: 680–686. 11022384 10.1038/sj.bdj.4800576

[5] Li X Tso SK Wang J Real-time tool condition monitoring using wavelet transforms and fuzzy techniques. IEEE Trans Syst Man Cybern Part C Appl Rev 2000; 30: 352–357.

[6] Watanabe I Ohkubo C Ford JP Cutting efficiency of air-turbine burs on cast titanium and dental casting alloys. Dental Mater 2000; 16: 420–425. 10.1016/s0109-5641(00)00038-5 10967191

[7] Ong FR Bouazza-Marouf K Drilling of bone: a robust automatic method for the detection of drill bit break-through. Proc IMechE, Part H: J Engineering in Medicine 1998; 212: 209–221. 10.1243/0954411981533999 9695640

[8] Ong FR Bouazza-Marouf K Evaluation of bone strength: correlation between measurements of bone mineral density and drilling force. Proc IMechE, Part H: J Engineering in Medicine 2000; 214: 385–399. 10.1243/0954411001535426 10997059

[9] Brett PN Baker DA Taylor R Controlling the penetration of flexible bone tissue using the stapedotomy micro-drill. Proc IMechE, Part I: J Systems and Control Engineering 2004; 218: 343–351.

[10] Brett PN Taylor RP Proops D An autonomous surgical robot applied in practice. In: Billingsley J Bradbeer R (eds) Mechatronics and machine vision in practice, vol. 5, 2008 Springer, pp. 261–266.

[11] Baker D Brett PN Griffiths MV A mechatronic drilling tool for ear surgery: a case study of some design characteristics. Mechatronics 1996; 6: 461–478.

[12] Lammie GA A comparison of the cutting efficiency and heat production of tungsten carbide and steel burs. Br Dent J 1951; 90: 251–259. 14830680

[13] Lammie GA A study of some different tungsten carbide burs. Dent Rec 1952; 72: 285–300.

[14] Norman DH A preliminary appraisal of an air-bearing handpiece. Br Dent J 1963; 114: 90–92.

[15] Henry EE Peyton FA A study of the cutting efficiency of dental burs for the straight handpiece. J Dent Res 1951; 30: 854–869. 14897981 10.1177/00220345510300061801

[16] Johnson GK Perry FU Pelleu GB Effects of four anticorrosive dips on the cutting efficiency of dental carbide burs. J Am Dent Assoc 1987; 114: 648–650. 3474268 10.14219/jada.archive.1987.0154

[17] Wirthlin MR Shklair IL Northener RA The performance of autoclaved high-speed dental handpiece. J Am Dent Assoc 1981; 103: 584–587. 7026643 10.14219/jada.archive.1981.0304

[18] Eames WB Reder BS Smith GA Ten high speed handpieces: evaluation of performance. Oper Dent 1979; 4: 124–131. 398978

[19] Wain EA Hesmondhalgh DE Fundamental design factors in rotary dental instrumentation. J Dent 1980; 8: 135–143. 6930387 10.1016/0300-5712(80)90030-5

[20] Newsome PRH Youngson CC Speed variability of air-driven motors in the self-threading pin technique. Br Dent J 1989; 166: 287–289. 2719888 10.1038/sj.bdj.4806809

[21] Harkness N Davies EH The cleaning of dental diamond burs. Br Dent J 1983; 154: 42–45. 6572060 10.1038/sj.bdj.4804985

[22] Taira M Wakasa K Yamaki M Comparison of rotational speeds and torque properties between air-bearing air-turbine handpieces. Dent Mater J 1989; 8: 26–34. 2700689 10.4012/dmj.8.26

[23] Taira M Wakasa K Yamaki M Effects of diamond grit sizes of the commercial dental diamond points on the weight load cutting of bovine enamel and glass ceramic typodont teeth. Dent Mater J 1990; 9: 173–180. 1966019 10.4012/dmj.9.173

[24] Taira M Wakasa K Yamaki M Dental cutting behavior of mica-based and apatite-based machinable glass ceramics. J Oral Rehabil 1990; 17: 461–472. 2231164 10.1111/j.1365-2842.1990.tb01417.x

[25] Ball JS Davidson CW Estimation of air turbine rotational speed under clinical conditions. Br Dent J 1962; 112: 243–248.

[26] Imbeniv J Kruzic J Marshall GW The dentin–enamel junction and the fracture of human teeth. Nat Mater 2005; 3: 229–232. 10.1038/nmat1323 15711554

[27] Cuy JL Mann AB Livi KJ Nanoindentation mapping of the mechanical properties of human molar tooth enamel. Arch Oral Biol 2002; 47: 281–291. 11922871 10.1016/s0003-9969(02)00006-7

[28] Poolthong S Determination of the mechanical properties of enamel, dentine and cementum by an ultra micro-indentation system. DDS, Clinical Science, Chulalongkorn University, Thailand, 1998, pp. 145–149.

[29] Habelitz S Marshall SJ Marshal GW Mechanical properties of human dental enamel on the nanometre scale. Arch Oral Biol 2001; 46: 173–183. 11163325 10.1016/s0003-9969(00)00089-3

